# An Instrumented Apartment to Monitor Human Behavior: A Pilot Case Study in the NeuroTec Loft

**DOI:** 10.3390/s22041657

**Published:** 2022-02-20

**Authors:** Stephan M. Gerber, Michael Single, Samuel E. J. Knobel, Narayan Schütz, Lena C. Bruhin, Angela Botros, Aileen C. Naef, Kaspar A. Schindler, Tobias Nef

**Affiliations:** 1 Gerontechnology & Rehabilitation Group, ARTORG Centre for Biomedical Engineering Research, University of Bern, 3012 Bern, Switzerland; michael.single@unibe.ch (M.S.); samuel.knobel@unibe.ch (S.E.J.K.); narayan.schuetz@unibe.ch (N.S.); lena.bruhin@unibe.ch (L.C.B.); angela.botros@unibe.ch (A.B.); aileen.naef@unibe.ch (A.C.N.); tobias.nef@unibe.ch (T.N.); 2 Department of Neurology, Inselspital, Bern University Hospital, University of Bern, 3012 Bern, Switzerland; kaspar.schindler@insel.ch

**Keywords:** instrumented apartment, neurodegenerative disorders, sensors, home-monitoring

## Abstract

For patients suffering from neurodegenerative disorders, the behavior and activities of daily living are an indicator of a change in health status, and home-monitoring over a prolonged period of time by unobtrusive sensors is a promising technology to foster independent living and maintain quality of life. The aim of this pilot case study was the development of a multi-sensor system in an apartment to unobtrusively monitor patients at home during the day and night. The developed system is based on unobtrusive sensors using basic technologies and gold-standard medical devices measuring physiological (e.g., mobile electrocardiogram), movement (e.g., motion tracking system), and environmental parameters (e.g., temperature). The system was evaluated during one session by a healthy 32-year-old male, and results showed that the sensor system measured accurately during the participant’s stay. Furthermore, the participant did not report any negative experiences. Overall, the multi-sensor system has great potential to bridge the gap between laboratories and older adults’ homes and thus for a deep and novel understanding of human behavioral and neurological disorders. Finally, this new understanding could be utilized to develop new algorithms and sensor systems to address problems and increase the quality of life of our aging society and patients with neurological disorders.

## 1. Introduction

The higher prevalence of certain age-associated neurological disorders (e.g., Parkinson’s, Alzheimer’s, multiple sclerosis, epilepsy), is in line with a steady increase of the average life expectancy [1]. With the progression of certain neurodegenerative disorders and the increasing number of older adults, the need for institutional care intensifies, which contrasts with the desire of most older adults and patients to live independently [2]. Oftentimes, after hospitalization, older adults or patients can no longer perform simple activities of daily living, and therefore, they are no longer able to live independently at home, and thus are dependent on institutionalized care (e.g., nursing home, retirement home). In particular, physiological and movement parameters when performing activities (e.g., cooking, standing up from a chair, sleeping) during day and night are an indicator of the onset of a disease, change in health status, and finally hinting whether someone needs immediate care [3]. The idea of using sensor-derived medical information in combination with the rapid evolution of assistive technology enables medical professionals to obtain real-time sensor-based information of older adults or patients at home, which has been gaining traction over the last years. However, there is a lack of systems that can accurately monitor activities of daily living at home over a prolonged time period [4]. Hence, there is a significant need for new sensor systems that are able to bridge the gap between the laboratories and older adults’ or patients’ homes to provide adequate care and to support independent living and aging and monitoring of people at home.

There are mainly three types of sensors that can be used for home-monitoring purposes. First, object sensors, are integrated into everyday objects to measure, for example, typing fluency [5] or the detection of steps by a smartphone [6]. Second, ambient sensors, mounted near older adults or patients, for example, to measure the location by passive infrared sensors [7], gait velocity by motion sensors [8], balance by low-cost cameras [9], or heart and respiration rate during sleep by pressure sensors [10]. Third, wearable sensors, attached to the patient’s body, for example, are used to measure gait by accelerometers [11] or mental health by pulse oximeter sensor [12]. However, while Schütz et al. have shown that the large majority of patients accept a dedicated wearable like a smartwatch, they do not like to use other wearable sensors, such as additional accelerometers or electroencephalogram (EEG) recording patches for an extended amount of time [7]. Therefore, the combination of object and ambient sensors is a promising approach to monitor patients over a prolonged period of time at home, for example, to detect falls [13].

Such in-home monitoring approaches and their impact on health have been investigated by Wang et al. and others. They have found that, while physiological functions cannot be unobtrusively measured with high accuracy, there is evidence that behavioral data, like ones present in a room, can be measured accurately [4,14,15]. Further, most systems focus on one specific and not on multiple outcomes at once, for example, using a specialized device to detect eye diseases [16] or wearables to detect functional fitness [17]. Furthermore, sensor systems have been found to be helpful in identifying adverse changes in behavior or vital signs prior to the occurrence of a life-threatening event (e.g., stroke, heart arrhythmia, obstructive sleep apnoea) [12]. However, there is currently no evidence that a multi-sensor home-monitoring system has a positive effect on health [14]. Potentially, sensor fusion, the combination of different types of sensors, might decrease the false alarm rate, and thus could support or even replace invasive tests and increase the comfort of patients [14]. 

Since sensor systems have the potential to increase efficiency and simplify the monitoring for medical professionals, several concept apartments like the KRIPIS Smart Home at CERTH/ITI have been introduced [18]. However, those sensor systems often do not combine basic sensors (e.g., accelerometer, radars, or depth cameras) and in parallel provide medically validated gold-standard devices, and thus have a hurdle to develop sensor systems suitable for medical applications under realistic conditions. 

Therefore, the focus of this pilot case study was on the development of a sensor system, installed in an apartment, based on basic technologies (e.g., infrared, radar) and validated medical gold-standard devices. We hypothesize that the system can unobtrusively record physiological and movement parameters of daily living activities performed during the day and night, allowing us to bridge the gap between laboratories and older adults’ or patients’ homes for the development and validation of new sensor systems.

## 2. Materials and Methods

### 2.1. The Instrumented Apartment

**Figure 1:**
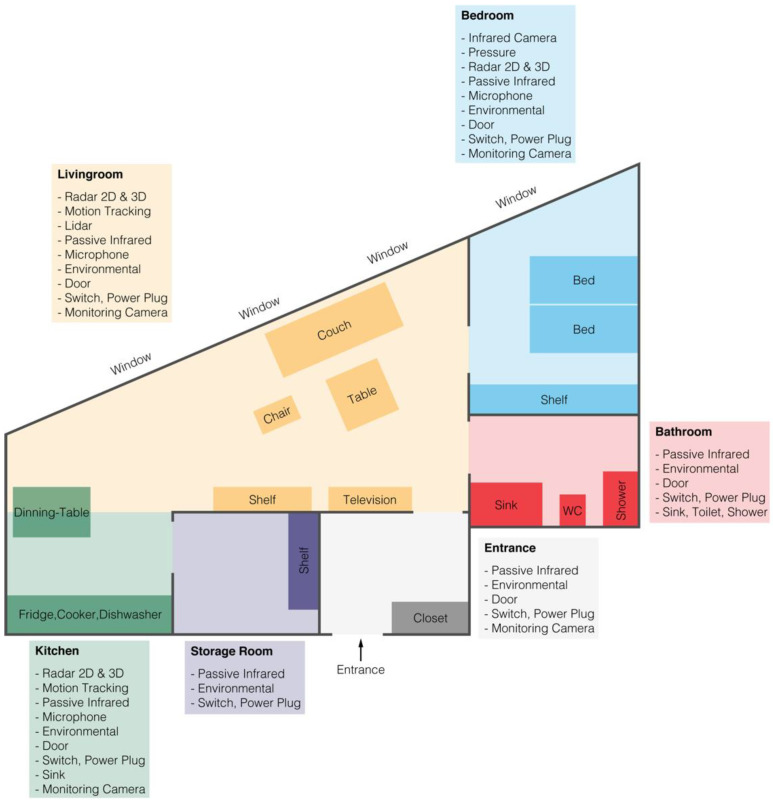
Layout of the instrumented apartment and markers of its installed sensors.

**Figure 2:**
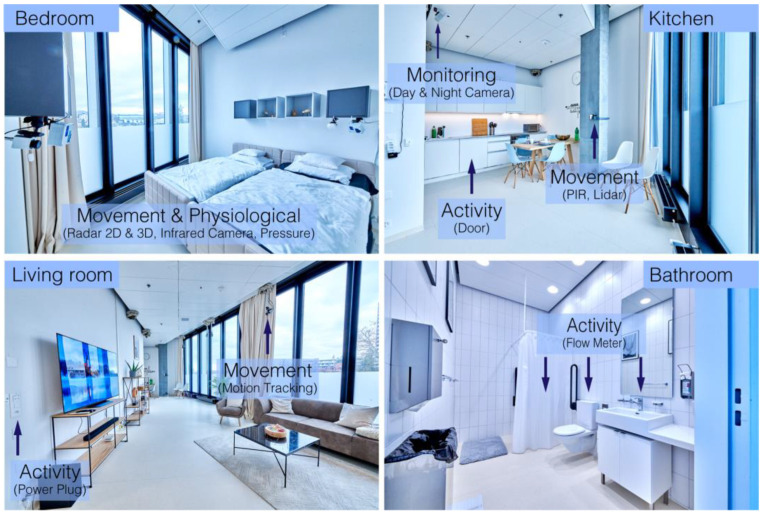
The instrumented apartment (NeuroTec loft): The installed sensors and their functions are indicated by blue shading.

The instrumented apartment, the NeuroTec Loft, has 3.5 rooms and is located within the SITEM (Swiss institute for translational and entrepreneurial medicine, University Hospital Bern, Inselspital, Switzerland) building and fulfills the requirements of a hospital room. Inside the apartment, unobtrusive contactless sensors were installed to monitor physiological and movement parameters and related activities of daily living (environment parameters) during the day and night. In addition, to monitor the experiment, cameras were installed, and wearables were provided (see Table 1, Figure 1 and Figure 2). All sensors follow the general safety requirements. Sensors with direct contact with the participant were FDA or CE approved. All sensors installed in the instrumented apartment were based on basic technologies, and for each technology a gold-standard medical device was available.

### 2.2. The Data Flow and Analysis

**Figure 3:**
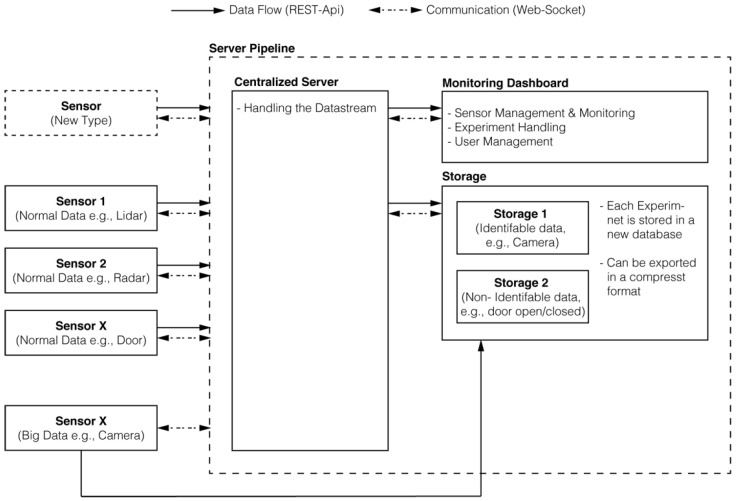
An illustration of the data acquisition monitoring system. Each box represents a particular physical entity in the pipeline, such as the sensors, the server, or the storage and all the arrows represent the possible communication and data-streams among those entities.

The measurement of participants and the corresponding data acquisition was realized by a customary built monitoring system, which follows the server-client design architecture (see Figure 3). Each single sensor device (e.g., radar sensors) communicates with a centralized server over a local (in our case 10 Gbps) network. The data flow from any sensor to the server is performed via HTTP (i.e., by relying on the REST paradigm), whereas the sensor control-flow is realized via streaming technologies (WebSockets). In order to ensure that the communication protocol is agnostic to the utilized sensor devices, a sensor-interface was developed. More precisely, this interface defines the structure and type of data contained in a sensor packet’s payload, how high-frequency sensors are supposed to aggregate their packets, and strategies addressing how network-related issues should be handled. All the acquired data is persisted in a database (MongoDB) and can be exported in a specified tabular format (i.e., Parquet), which is then persisted on long-term storage (network-attached storage, NAS). Sensors that produce too large chunks of data (e.g., the motion tracking system) can also directly persist their data on the NAS. To simplify the process of managing the pipeline, the definition of experiments, the administration of sensors (e.g., active/stopped, recording), and the handling of the database, a user interface, the so-called dashboard, has been implemented. Ethical requirements, such as storing video-recordings, not alongside other participant identifying data, could be realized by storing certain database chunks on different NAS devices.

The exported data was analyzed using R and Python. Due to being a pilot case study, only descriptive analysis was performed. The correctness of single events (e.g., using the microwave or flushing the toilet) were double checked by the video recordings, however, due to privacy, were not shown.

### 2.3. Participant and Experiment

The case study was approved by the Intuitional Review Board of the Canton of Bern, Switzerland (KEK no. 2020-02771). The participant signed informed consent, was briefed, and the Declaration of Helsinki was followed. The inclusion criteria were age over 18 years, no neurological disorder, and not infected by multi-drug-resistant bacteria or incontinent. The participant, male, 32 years old, was recruited by word of mouth at the University of Bern in August 2021 and spent an evening, night, and morning in the instrumented apartment. The participant was not given any task to perform or schedule to follow and thus, behaved as he would at home, except for living in the instrumented apartment. The only restriction was that he was not allowed to have any visitors during the stay. After his stay, the participant was asked verbally about his experience in the instrumented apartment. Further, a long-term test was performed to measure consistency and data loss of the developed sensor system.

During the experiment, all contactless sensors recorded the activities of daily living. No wearable sensors were integrated into the experiment (see Table 1).

## 3. Results

**Figure 4:**
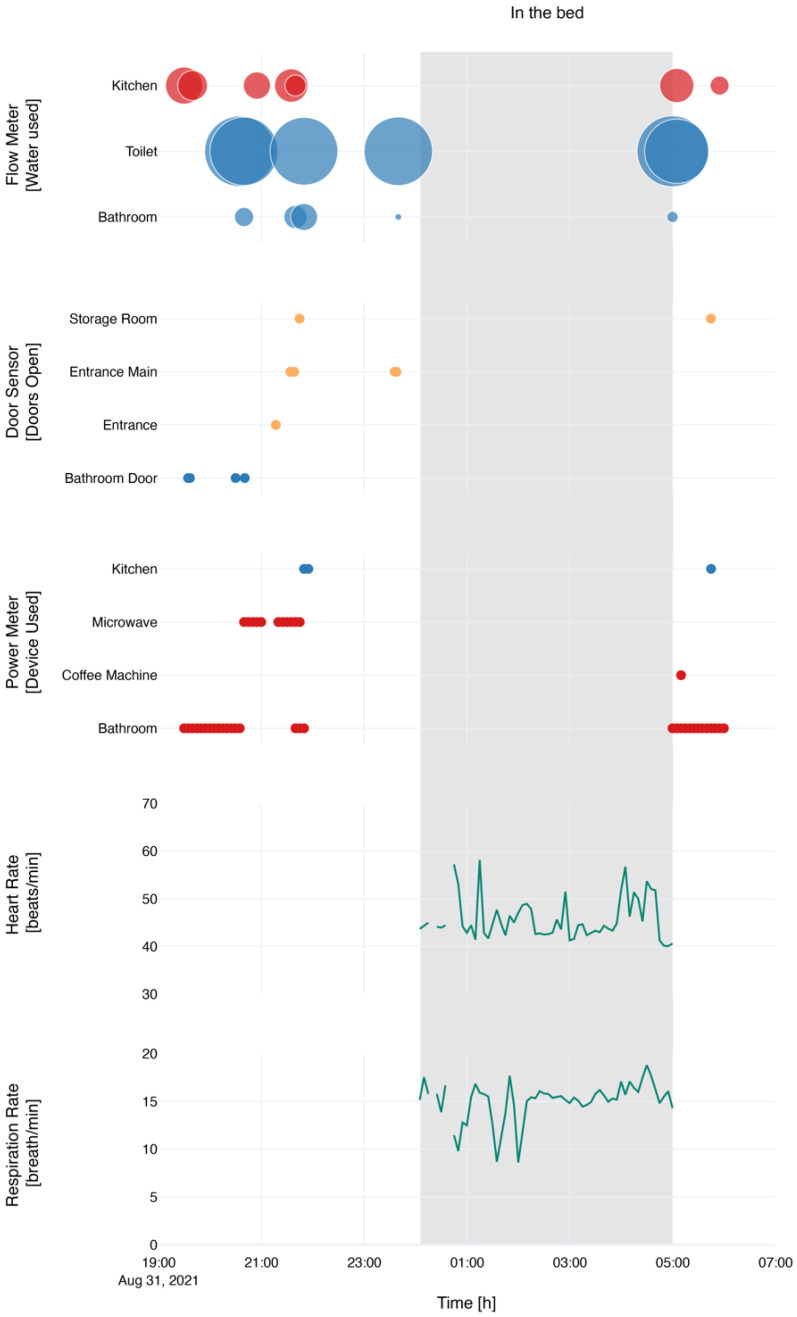
Selection of sensors during the stay of the participant. Tasks colored in red are related to the kitchen, in blue to the bathroom, in orange to the storage room and entrance area, and in green to the night. The flow meter showed the relative water usage during the stay, whereas the power meter and door sensor showed whether a door was opened, or a device or power plug was used.

The system measured the first events at 19:00 and stopped measuring at 07:00 the next day, resulting in a 12-h stay. During that time, the participant had dinner using the microwave oven, spent time in all the rooms, slept, and had breakfast before leaving the instrumented apartment as illustrated in Figure 4. 

**Figure 5:**
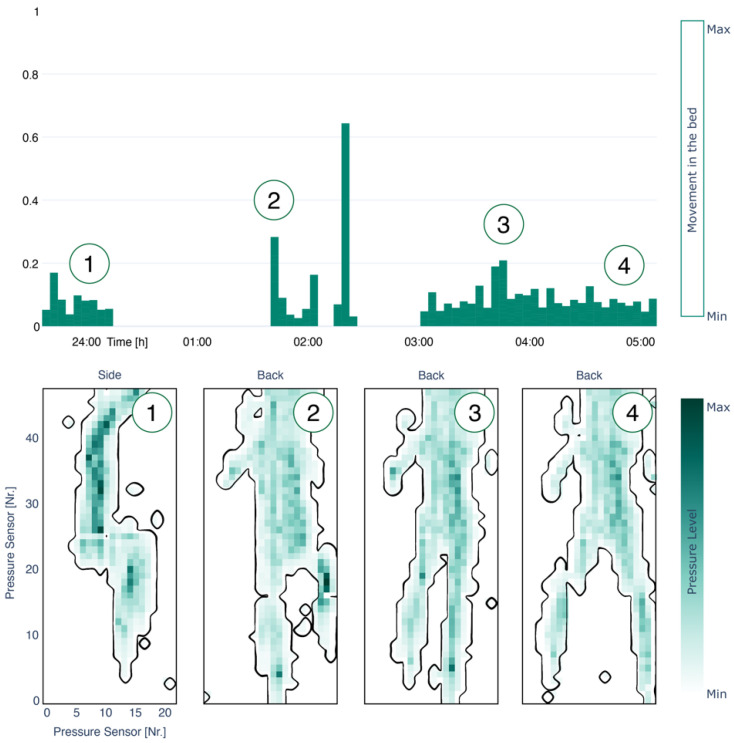
Top panel: The bar plot (i.e., movement in the bed) illustrates the activity of movement during sleep (i.e., the normalized sum of the difference between pressure level maps). The strongest movements were present at twenty past two.

During the night a ballistocardiography sensor, installed under the mattress, was running, resulting in the following insights. The participant spent approximately five hours in bed, had one bed exit, 96 toss and turns, and a mean heart rate of 47.70 ± 4.35 beats/min and respiration rate of 15.01 ± 2.05 breath/min. As can be seen in Figure 5, the participant first slept on his side and then turned to his back. 

Further, measured by environmental sensors, the mean temperature during the stay was 24.75 ± 1.43 °C, mean humidity 39.47 ± 2.53% and the red-light spectrum 925.78 ± 1006.76, blue = 875.90 ± 485.74, green = 809.92 ± 601.62, clear = 1089.49 ± 825.42. Overall, 717.46 units of water were used. The used water was highest for the toilet 420.21 (M = 70.03 ± 2.45 pulse counts) followed by the kitchen sink 207.29 (M = 17.99 ± 8.55 pulse counts) and the bathroom sink 89.96 (M = 29.61 ± 7.01 pulse counts). The highest power consumption was in the bathroom 6.26 kW, followed by the kitchen 1.14 kW, the microwave 0.97 kW and the coffee machine 0.43 kW. During the stay, the kitchen trash was opened nine times, the dishwasher once, and four cupboard doors were opened. No other closet doors or power plugs were used. However, the bathroom door was opened and closed three times, the main entrance door three times, and the storage room door once. Furthermore, during the stay, the microwave and the coffee machine were used.

Overall, no sensor malfunction occurred. Furthermore, during the long-time test of 1362 h, in total 84,692,300 data packages were sent, whereas 868 did not arrive at the server pipeline (i.e., data loss). Further, as seen in Table 2, the system in the long-time test was running stable and had a high consistency.

In the interview, the participant did not report any negative experience, on the contrary, he reported enjoying his stay in the apartment. However, he stated that certain adjustments to being monitored were needed. 

Lower panel: The four illustrations (i.e., pressure level) show the position of the participant over time during sleep, while dark green indicates high pressure on the body, and white no pressure. At the beginning of sleep, he was on his side and turned afterward to his back. In total, the system has 22 × 48 sensors integrated. 

## 4. Discussion

In this pilot case study, we demonstrated a new system that is based on sensor technologies to monitor movement, physiological parameters and related activities of daily living during day and night. In line with our hypothesis, the system was able to accurately measure and identify different daily actives.

The first finding was that the proposed system is able to measure on a multimodal level, the movement and physiological and thus relevant medical parameters over a prolonged period of time. During the stay, the behavior of a healthy participant was monitored. In addition, the participant did not state any negative experience, which confirms that the system was unobtrusive in measuring movement, and physiological and environmental parameters, which has been made possible by using ambient and object sensors [14,19].

The second finding was the capability of the system to assess the movement and physiological parameters synchronized on a multidimensional scale. This means, for example, the movement in the bed or the position in the living room can be assessed by a different sensor simultaneously. Its multidimensional scale and the comparison to the gold standard and participants in a standardized environment offer the opportunity to validate new sensor systems and to replace expensive systems or increase the measurement efficiency and accuracy [20]. Overall, the monitored data of the presented system allows for assessing phenotypes of certain diseases and the development and validation of new medical sensor systems, modalities or settings of a sensor (e.g., stimulation pattern for deep brain stimulation), and diagnosis tools.

However, one important aspect of monitoring is whether it is potentially beneficial for the participant and whether privacy is maximized [21]. To increase privacy aspects, it is crucial to understand the functionality of the different sensors, meaning does the assessed outcome answer the research questions or does the data assess information that is not needed (e.g., camera or microphone data). To address this concern, the developed sensor system offers the possibility to combine sensor data streams and to offer insight on how unobstructive sensors can comfort participants while preserving privacy. 

In order to prevent the change of response or behavior while being monitored (i.e., the Hawthorne-Effect), participants should feel as comfortable during their stay as they do at home [22]. Therefore, whenever possible, all sensors were shielded, in a way that participants did not even detect them. A solution to that problem could be to accustom a participant to the setting of being monitored. This, however, would result in discarding the first few hours of recording.

Even though we could confirm our hypothesis in this pilot case study, large studies are needed to find generalizable results that can be transferred to the population of neurological patients and to home settings. An additional limitation is that not all sensor systems are validated against the gold standard; therefore, in the next step, we will investigate the accuracy of measured (i.e., the difference between two single sensors) vitals, movement, and environment parameters by applying a noninferiority design. 

In this case, three studies are planned to show the potential of the developed sensor system. First, a clinical study to increase the quality of life in Parkinson’s patients, where the stimulation parameters of a deep brain stimulator are adapted to the activities of daily living of the patient. Second, a clinical study to assess the severity of multiple sclerosis compared to medical assessment in the clinics based on patient activities of daily living. Third, the development of new algorithms to assess gait parameters based on radar technology.

## 5. Conclusions

In the pilot case study, a sensor system to measure multimodal data, movement, physiological and environmental parameters was presented. The sensor system has great potential to bridge the gap between laboratories and older adults’ or patients’ homes in order to advance science in terms of human behavioral and neurological disorders. Finally, the sensor system allows the development and validation of new algorithms and sensor systems to address medical problems and increase the quality of life of our aging society and patients with neurological disorders.

## Figures and Tables

**Table 1:** A list of all the installed sensors in the instrumented apartment and their properties. Notice that a * marking stands for a gold-standard device, which is used as a reference system.

	Primary Parameters	Technology	Signal Format	Time		Contactless		Gold *	Device
				Day	Night	Yes	No		
*Physiological*	Heart rate, Breathing rate	Pressure Sensor	Piezoelectrical		X	X			EMFIT, Finland
	Heart rate, Breathing rate	Radar Sensor 2D & 3D	Electromagnetic		X	X			Somnofy, Norway
	Blood pressure, Heart rate, Breathing rate	Infrared Camera	Temperature Image		X	X			Optris, Germany
	Skin resistance	Galvanic Sensor	Electrodermal activity				X		Empatica, United States of America,
	Heart rate, Breathing rate, Blood pressure, Oxygen saturation	Mobile polysomnography (Pleth- Sensor, Electrocardiogram (ECG))	Reflection of light, Electrical activity	X	X		X	X	Somnomedics, Germany
*Movement*		Pressure Sensor	Piezoelectrical		X	X			SensingTex, Spain
		Radar Sensor 2D & 3D	Electromagnetic		X	X			Somnofy, Norway, RFbeam, Switzerland
		Accelerometer	Rate of change of velocity	X	X		X		Axivity, United Kingdom
		Gyroscope	Orientation and angular velocity	X			X		GaitUp, Switzerland
		Motion Tracking System	Video Image	X		X		X	Qualisis, Sweden
		Lidar-, PIR-sensors	Reflection of light	X		X			Hokuyo, Japan
*Environmental*	Speech, Ambient noise	Microphone	Sound	X	X	X			Sennheiser, Germany
	Illuminance, Humidity, Temperature	Environmental Sensor	Light, Humidity, Temperature	X	X	X			Rohm, Japan
	Doors open and closed	Door sensor	Magnetic	X		X			Domosafety, Switzerland
	Devices on and off	Switch, Power plug	Electrical current	X		X			Shelly, United States
	Water on and off	Sink & Shower Senor	Water flow	X		X			Swissflow, Netherlands

**Table 2:** Consistency of the server pipeline and the different sensors during the long-term test (i.e., 1362 h). Each data package consisted of 100 data entries. *n* = number of data packages sent. Mean = Time between the previous and next data package of the same sensor type arriving at the server pipeline, q25 = 25% quantile, q75 = 75% quantile.

Sensor Type	*n*	Mean [ms]	Std [ms]	q25 [ms]	q75 [ms]
Flow Meters	2,597,253	1087.38	100,928.71	1000	1000
Door Sensors	2,072,136	1946.29	36,916,172.47	1000	2000
Power Meters	2,274,511	666.66	47,101,113.59	333.33	1000
Environmental Sensors	935,192	2393.46	1362.13	1000	4000
Bed Pressure Sensors	566,748	2306.95	913,374.84	1000	1000

## Data Availability

The data is not publicly available due to Swiss privacy regulations.
